# Hepatitis C Virus and Hepatocellular Carcinoma

**DOI:** 10.3390/biology2010304

**Published:** 2013-01-30

**Authors:** Tatsuo Kanda, Osamu Yokosuka, Masao Omata

**Affiliations:** 1 Department of Gastroenterology and Nephrology, Graduate School of Medicine, Chiba University, 1-8-1 Inohana, Chuo-ku, Chiba 260-8670, Japan; E-Mail: yokosukao@faculty.chiba-u.jp; 2 Yamanashi Hospitals (Central and Kita) Organization, 1-1-1 Fujimi, Koufu-shi, Yamanashi 400-8506, Japan; 3 University of Tokyo, 7-3-1, Hongo, Bunkyo-ku, Tokyo 113-8655, Japan

**Keywords:** androgen receptor, apoptosis, gender difference, hepatitis C virus, hepatocellular carcinoma, inflammation

## Abstract

Hepatitis C virus (HCV), a hepatotropic virus, is a single stranded-positive RNA virus of ~9,600 nt. length belonging to the *Flaviviridae* family. HCV infection causes acute hepatitis, chronic hepatitis, cirrhosis and hepatocellular carcinoma (HCC). It has been reported that HCV-coding proteins interact with host-cell factors that are involved in cell cycle regulation, transcriptional regulation, cell proliferation and apoptosis. Severe inflammation and advanced liver fibrosis in the liver background are also associated with the incidence of HCV-related HCC. In this review, we discuss the mechanism of hepatocarcinogenesis in HCV-related liver diseases.

## 1. Introduction

Hepatitis C virus (HCV) infection affects 3–4 million people every year and ~170 million people are chronically infected with this virus [1]. HCV infection causes acute and chronic hepatitis, cirrhosis and hepatocellular carcinoma (HCC) [2]. Although peginterferon and ribavirin together with or without telaprevir or boceprevir are currently used for HCV infection, a significant number of infected individuals do not respond to this treatment [3,4]. As a result, more than 350,000 people die every year from HCV-related liver diseases such as cirrhosis and HCC [1].

HCV is a positive-sense single-stranded RNA virus belonging to the *Flaviviridae* family. The HCV genome is approximately 9,600 nt. in length and consists of a 5' nontranslated region (5' NTR), a single open reading frame that encodes a polyprotein precursor of about 3,000 amino acids, and a 3' NTR. Both structural (core, E1, E2, and p7) and nonstructural proteins (NS2, NS3, NS4A, NS4B, NS5A, and NS5B) are cleaved from the single open reading frame by both viral and host proteases [5,6]. The HCV genome also has an internal ribosomal entry site (IRES) that can promote 5'-end-independent initiation of RNA translation [7].

HCV proteins are reported to interact with host-cell factors that are involved in cell cycle regulation, transcriptional regulation, cell proliferation and apoptosis [8]. Severe inflammation and advanced liver fibrosis in the liver background are also associated with the incidence of HCV-related HCC [9,10]. In this review, we discuss the mechanism of hepatocarcinogenesis in HCV-related liver diseases.

## 2. Signaling Pathways Affected by HCV Proteins

### 2.1. Signaling Pathways Involved in Hepatocarcinogenesis

**Figure 1:**
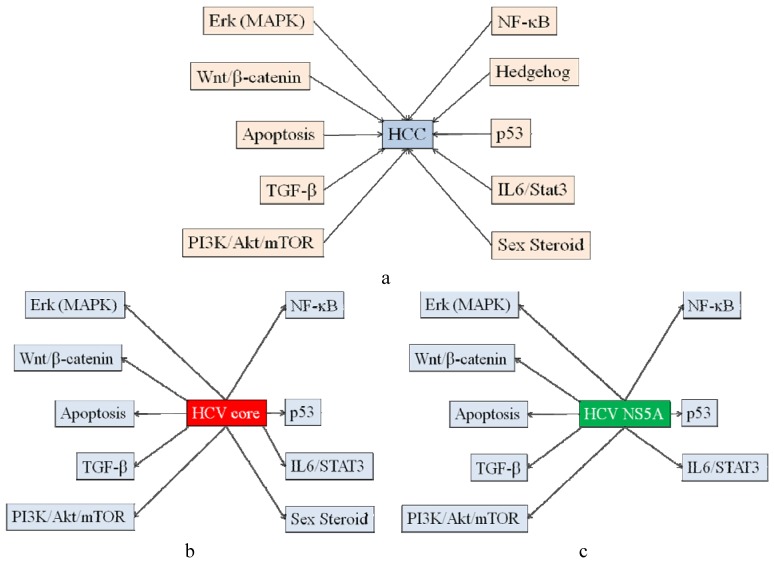
Signaling pathways affected by Hepatitis C virus (HCV) proteins. (**a**) Signaling pathways involved in hepatocarcinogenesis. (**b**) Signaling pathways affected by HCV core protein. (**c**) Signaling pathways affected by HCV NS5A protein.

The molecular pathways to hepatocarcinogenesis involve many signaling pathways such as extracellular signal-regulated kinase [Erk: mitogen activated protein kinase (MAPK)], Wnt/β-catenin, apoptosis, transforming growth factor-beta (TGF-β), phosphoinositide-3-kinase(PI3K)/Akt, mammalian target of rapamycin (mTOR), nuclear factor kappa-light-chain-enhancer of activated B cells (NF-κB), Hedgehog, p53, cytokine and sex steroid pathways [11] (Figure 1A). Unique pathways do not seem to contribute to hepatocarcinogenesis. HCV is an RNA virus, and it is thought to be unable to integrate its genome into the host genome, in contrast to hepatitis B virus (HBV) or human immunodeficiency virus (HIV). However, HCV proteins and the interaction between them and host proteins mainly contribute to the viral oncogenic processes [8,12].

### 2.2. Signaling Pathways Affected by HCV Core Protein

HCV core protein is a basic protein that is thought to comprise the nucleocapside of HCV, and its size is 17–23 kDa [8]. Ray *et al*. [13] reported that HCV core protein has oncogenic potential. That is, HCV core protein cooperates with ras and transforms primary rat embryo fibroblasts to tumorigenic phenotype. HCV core protein can activate the Erk (MAPK) signaling pathway [13,14,15,16,17,18], upregulate Wnt/β-catenin pathway [16,19], suppress apoptosis pathway [20,21,22], and activate TGF-β [23,24], PI3K/Akt/mTOR [25,26,27,28,29], NF-κB [30,31,32], p53 [33,34,35], IL-6/Stat3 [36,37,38] and androgen receptor (AR) pathways [39] (Figure 1B). Through these pathways, cell growth, differentiation, apoptosis, transcription and angiogenesis might be regulated by HCV core protein.

HCV core might represent a novel type of Raf-1 kinase-activating protein through HCV core-14-3-3 protein interaction and contribute to hepatocyte growth regulation [14]. HCV core can directly activate the MAPK cascade and prolong its activity in response to mitogenic stimuli, contributing to the neoplastic transformation of HCV-infected liver cells [16]. HCV core protein promotes proliferation of human hepatoma cells by activation of MAPK pathway via up-regulation of TGFα transcription by activation of NF-κB [18]. HCV core interacts with NF-κB signaling pathway [30,31,32]. HCV core protein promotes hepatocyte growth at least partly through transcriptional upregulation of growth-related genes, and in particular Wnt [19].

HCV infection is associated with hepatocyte cell death such as apoptosis [20,21,22] and autophagy [40]. The ability of core protein to inhibit the TNF-mediated apoptotic signaling pathway may provide a selective advantage for HCV replication [20,22]. HCV core also inhibits Fas-mediated apoptotic cell death via a mechanism dependent on the activation of NF-κB [21]. HCV core protein expression may directly upregulate TGF-beta 1 transcription in hepatocytes [23]. HCV core protein activates Akt through the Ras/PI(3)K pathway [25,26,27,28,29]. HCV core protein may also play an important biological role in the promotion of cell growth by repressing p53 transcription [33]. Further, HCV core protein acts as an effector in the promotion of cell growth by repressing p21 transcription [34]. HCV core protein may directly influence the various p73 functions, playing a role in HCV pathogenesis [41] (Table 1).

**Table 1:** HCV proteins core and NS5A, and apoptosis.

HCV protein	Host protein interacted with HCV protein [Reference]	Action
core	TNF receptor-1 [42], NF-κB [21], TRADD, TRAF [43], pRb [44], p73 [41], 14-3-3epsilon [45], Hsp60 [46], Mcl-1 [47]	pro-apoptosis
core	NF-κB [48], p38 MAPK [49], Bcl-x [50], p53 [51], p73 [41], Inhibitor of caspase-activated DNase [52], p21, Bcl-2 [53], Apaf-1, E2F1 [54], Grp78/Bip, Grp94 [55], PML [56], Stat3 [37], cFLIP [22], DR5 [57]	anti-apoptosis
NS5A	Bax [58],	pro-apoptosis
NS5A	PKR, eIF-2alpha [59], TRADD [60], p53 [61,62], Grb2 [63], PI3K [63,64], NF-κB [65], Bin1 [66], FKBp38 [67,68], calpain cystein protease, Bid [69], Kv2.1 [70], TLR4 [71]	anti-apoptosis

HCV core-induced Stat3 activation also plays an important role in hepatocarcinogenesis [35,36,37,38]. HCV core protein enhances vascular endothelial growth factor (VEGF) expression and facilitates angiogenesis in the presence of AR and acts as a positive regulator in AR signaling [39].

### 2.3. Signaling Pathways Affected by HCV NS5A Protein

HCV NS5A exists as two phosphoproteins, p56 and p58, phosphorylated at serine residues after the mature protein is released from the polyprotein [72]. HCV NS5A plays a critical role in the perturbation of MAPK signaling pathways in HCV-infected hepatocytes [73]. HCV NS5A activates β-catenin signaling cascades by increasing its stability [74], and HCV NS5A protein interacts with β-catenin and stimulates its transcriptional activity in a PI3K-dependent fashion [75,76]. HCV NS5A protein protects against LPS [71] or TNF-α-mediated apoptotic cell death [60,77] (Table 1). Further, HCV NS5A activates TGF-β signaling [78,79], induces EMT and participates in oncogenic transformation of primary hepatocyte precursors and in mouse hepatic progenitors along with cooperative oncogene H-RasV12 [80]. HCV NS5A also activates PI3K/Akt/mTOR pathway [63,64,68,81] and NF-κB signaling [82,83,84,85]. In addition, HCV NS5A physically associates with p53 and regulates p21/waf1 gene expression in a p53-dependent manner [86,87,88]. HCV NS5A-mediated Stat3 activation requires co-operation of Jak1 kinase [82,89] (Figure 1C). HCV NS5A induces a range of liver pathology including HCC in transgenic mice [90], although HCV NS5A transgenic animals are also valuable models of HCV immunopathology [91]. HCV probably does not induce HCC in transgenic mice [4,60,88,91,92].

### 2.4. Signaling Pathways Affected by other HCV Proteins

Several studies have reported that cell transformation was induced by HCV NS3 proteins [93,94]. JNK activation is essential for the stimulation of HCV NS3-mediated cell growth [95]. The *N*-terminal portion of NS3 forms a complex with the tumor suppressor p53 and suppresses actinomycin D-induced apoptosis [96]. There have been several reports regarding other HCV proteins interacting with host proteins and suggesting that these might lead to hepatocarcinogenesis [97,98,99].

### 2.5. HCV-Associated Inflammation Induces HCC

Takano *et al*. prospectively investigated the incidence of HCC in 124 cases of hepatitis C and found that HCC occurred in 13 chronic hepatitis C cases, consisting of 12 cirrhotic livers and only 1 non-cirrhotic liver [9]. A recent study with transient elastography also supports this observation [100]. Several lines of evidence [10,101] support the concept that HCV-associated inflammation causes HCC. Chronic HCV infection represents the increases in endoplasmic reticulum (ER) stress and oxidative stress [102,103,104,105,106,107]. The accumulation of unfolded proteins in ER causes ER stress and the unfolded protein response (UPR), mediated by the ER-resident stress sensors ATF-6, IRE1, and PERK, and the genes involved in the control of diffuse processes such as liver proliferation, inflammation, and apoptosis were significantly induced in chronic hepatitis C patients [102,103,104,105,106]. Increased hepatic iron deposition is common in HCV-infected patients. Excessive iron is known to generate ROS within hepatocytes, causing mutagenic lesions such as 8-hydroxy-2'-deoxyguanosine (8-OHdG) in DNA. Long-term iron depletion by phlebotomy for chronic hepatitis C patients is a promising modality for lowering the risk of progression to HCC [108,109,110,111]. The prevalence of steatosis in HCV-infected patients is ~70%. Age at liver biopsy, body mass index (BMI) and duration of HCV were independent risk factors for increased fibrosis in HCV patients. Steatosis as a risk factor for fibrosis is evident in genotype-1 [112]. Nieminen *et al*. [112] recommended that the degree of steatosis be evaluated in addition to fibrosis and inflammation activity. It seems important that activating survival genes within cancer cells and inflammation-promoting genes in components of the tumor microenvironment may be important for the development of HCC [113]. Further studies will be needed for clarification.

## 3. Conclusions

**Figure 2:**
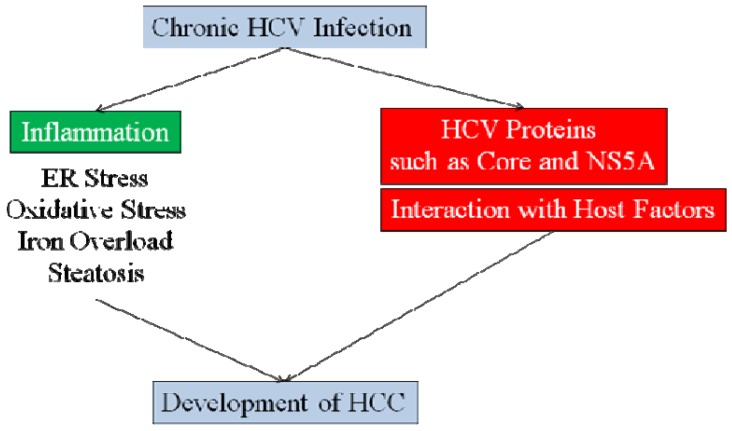
Development of hepatocellular carcinoma (HCC) after HCV infection.

HCV is a major risk factor for the development of HCC and there is increasing experimental evidence to suggest that the virus plays a direct role in neoplastic transformation. We reviewed the literature regarding two individual proteins of HCV, namely core and NS5A, and their role in the pathogenesis of HCC through perturbations of cellular pathways, in addition to their immunopathological effects of chronic inflammation. Inflammation as well as ER stress, oxidative stress, iron overload and steatosis in hepatocytes are also important factors (Figure 2). Not only eradication of HCV but also corrections of these factors play important roles in the prevention of the development of HCC.
